# Impairment of the Antibody-Dependent Phagocytic Function of PMNs through Regulation of the FcγRs Expression after Porcine Reproductive and Respiratory Syndrome Virus Infection

**DOI:** 10.1371/journal.pone.0066965

**Published:** 2013-06-25

**Authors:** Bo Wan, Songlin Qiao, Peng Li, Qianyue Jin, Yunchao Liu, Dengke Bao, Mingyang Liu, Yinbiao Wang, Gaiping Zhang

**Affiliations:** 1 College of Animal Science and Veterinary Medicine, Jilin University, Changchun, China; 2 Key Laboratory of Animal Immunology of the Ministry of Agriculture, Henan Provincial Key Laboratory of Animal Immunology, Henan Academy of Agricultural Sciences, Zhengzhou, China; The Ohio State University, United States of America

## Abstract

Porcine reproductive and respiratory syndrome (PRRS) is identified as one of the most important etiological agents in multifactorial respiratory disease of swine and can predispose pigs to secondary infections by other pathogens, usually bacteria. To understand the mechanism for an increased susceptibility to secondary bacterial infections, we investigated the antibody-dependent phagocytosis behaviour and killing ability of PMNs after infection by PRRSV strains BJ-4 or HN07-1. PMN’s antibody-dependent phagocytosis and their ability to kill *E.coli* were both noticeably decreased following PRRSV infection, in particular with the highly pathogenic strain HN07-1. As the change in this function of the PMNs may reflect a variation in the expression of FcγRs, the expression profiles of the activating and the inhibitory FcγRs were examined. We found that RNA expression of the inhibitory receptor FcγRIIB was up-regulated post-infection, and this was greater after infection with the more virulent PRRSV strain HN07-1. The activating receptor FcγRIIIA RNA expression was on the other hand inhibited to the same extent by both PRRSV strains. Neutralizing antibody titers post-infection by PRRSV strains BJ-4 or HN07-1 were also detected. All of the pigs in infection groups showed viraemia by the end of the study (56 DPI). These observations may help to understand the mechanism of increased susceptibility to secondary bacterial infections following PRRSV infection.

## Introduction

Porcine reproductive and respiratory syndrome virus (PRRSV), a member of the family *Arteriviridae* of non-segmented, positive-sense, single-stranded RNA viruses, was first isolated as the cause of porcine reproductive and respiratory syndrome (PRRS) in 1991 in Europe [1]–[3]. PRRS is characterized by severe respiratory disease in young pigs and reproductive failure in sows. It has become endemic in countries with high levels of pig rearing and causes a considerable economic loss each year [4]–[6].

PRRS was also identified as one of the most important etiological agents in multi-factorial respiratory disease of swine and can predispose pigs to secondary infections by many kinds of pathogens, usually bacteria. Many researchers have focused on studying the increased susceptibility to secondary bacterial infections after PRRSV infection. Associations were calculated between PRRSV and the other etiological agents, the results proved that pigs were predisposed to infection by bacteria including *Steptococcus suis, Haemophilus parasuis* and *Mycoplasma hyopneumoniae* [7]–[11]. A study on this indicated that virulent PRRSV strain predisposed pigs to *S. suis* infection more stronger than the attenuated vaccine strain of PRRSV [12], [13].

Recent studies suggested several possible explanations for the increased susceptibility to secondary bacterial infections following PRRSV infection. Decreased functioning of macrophages from PRRSV-infected pigs has been found. Thus at 7 days following PRRSV infection, alveolar macrophages had a decreased uptake of opsonized *H. parasuis* and decreased superoxide anion production; at 9 days there was increased intracellular survival of *H. parasuis* along with decreased superoxide anion production [14].

Polymorphonuclear leukocytes (PMNs) play a crucial role in the primary immunological defense against infectious agents by clearing bacteria through host innate receptors [15]–[18]. They have several well-established functions including the phagocytosis of opsonized particles and the production of reactive oxygen and nitrogen species in the killing of foreign target cells [19], [20]. PMNs interact with opsonized immune complexes through Fcγ receptors (FcγRs), activating and inhibitory receptors which bind the Fc domain of immunoglobulin G (IgG) [21]. Fc receptors fall into two categories: the activating and the inhibitory, which respectively transmit their signals via ITAM or ITIM terminal sequences [21], [22]. It is suggested that the activating receptor FcγRIIIA and inhibitory FcγRIIB have evolved as a paired antagonistic signaling system, allowing changes in their individual regulated expression levels to alter the overall stimulus induced by IgG immune complexes [23]–[27]. We hypothesize that the variations of FcγRs on PMN following PRRSV infections may reflect the function of PMNs in defense against infectious agents, and then contribute to secondary infections. Following PRRSV infection, the antibody-dependent phagocytosis and ability to kill *E.coli* of PMNs and the expression of FcγRs were investigated in an attempt to provide a further understanding of the potential reason for the increased susceptibility to secondary bacterial infections.

## Materials and Methods

### Virus Strains

Two PRRSV strains, BJ-4 and HN07-1, were used in this study. The BJ-4 strain (typical North American strain) was isolated in 1996 in China (GenBank: AF331831) and supplied by Dr. Hanchun Yang from China Agricultural University. The HN07-1 strain (a highly pathogenic North American strain) was isolated in Henan province by our group (GenBank: HQ025966) [28]. Both PRRSV strains were propagated on Marc-145 cells, and maintained in Dulbecco’s modified Eagle’s medium (DMEM) (Invitrogen) supplemented with 10% fetal bovine serum (FBS) (Sigma) at 37°C under 5% CO_2._

### Management of Pigs

Conventional Large White-Duroc crossbred piglets (n = 15), weaned at 35–40 d of age, were confirmed as seronegative for antibodies to PRRSV, PRV, CSFV and PCV2 with commercial Enzyme-Linked Immunosorbent Assay (ELISA) kits for antibody detections and with RT-PCR or PCR for viral nucleic acid detection.

Fifteen piglets were randomly assigned to one of three treatment groups (n = 5), two infection groups (BJ-4 or HN07-1) and a mock-infected control group. Every group was raised separately in three isolation rooms with individual ventilation. After an additional week for acclimatization, the piglets in the infection groups were inoculated intranasally (IN) and intramuscularly (IM) with 10^5^ TCID_50_ of PRRSV HN07-1 or BJ-4 respectively. Piglets of the mock group were inoculated with an equal volume of DMEM. Serum samples were collected throughout the experimental period for harvesting of PMNs, serum cytokine assay, antibody detection and flow cytometric (FCM) analysis (BD FACSCalibur).

The animal study proposal was authorized and supervised by the Institutional Animal Care and Use Committee (IACUC) of the Key Laboratory of Animal Immunology of the Ministry of Agriculture of China with the permit number: IACUC2011-016. All pig experimental procedures were performed in accordance with the Regulations for the Administration of Affairs Concerning Experimental Animals approved by the State Council of People’s Republic of China.

### PMNs Isolation

Porcine PMNs were isolated from heparinised freshly-drawn blood by Ficoll-HyPaque density (concentration 1×10^6^ cells/ml) gradient centrifugation followed by hypotonic lysis as previously [29], [30]. Briefly, the blood was left at room temperature for 30 min in 10 mL disposable tubes, and the leukocyte-rich buffy coats were taken. After centrifugation at 1500×g for 8 min on Ficoll-Paque, the pellet containing erythrocytes and PMNs were obtained. Erythrocytes were removed by hypotonic lysis, and PMNs were resuspended at a final concentration of 1×10^6^ cells/ml DMEM medium for the later experiments. Cell viability was approximately 98% as assessed by Trypan Blue staining.

### Quantification of Antibody-dependent Phagocytosis by PMNs

Polystyrene beads, 3 µm diameter, were opsonized by incubation with 10 mg/ml bulk porcine IgG (Sigma) in PBS with shaking for 1 h at 37°C. For scoring the phagocytic index, 1 ml of PMN cells (concentration 1×10^6^ cells/ml) were incubated with 100 µl of beads (concentration 1×10^7^ beads/ml) for 40 min at 37°C in a tissue culture incubator. The cells were washed three times with cold PBS, fixed in 100 µl of 4% paraformaldehyde, and mounted. Phagocytosis was quantified in terms of phagocytic index (internalized particles per 200 cells) [31].

### Immune Complex Adhesion and Uptake Assays

To make the immune complexes, a solution of 10 mg/ml FITC-conjugated ovalbumin (Sigma) was incubated with 200 µg/ml porcine polyclonal anti-ovalbumin antibody (Sigma) (OVA/anti-OVA ratio 1∶1) at 37°C for 1 h. 200 µL ovalbumin-Ig complexes were allowed to bind to cells (concentration 1×10^6^ cells/ml) for 30 minutes on ice. Excess ovalbumin-Ig complex was removed by washing three times with PBS. The percentage of PMNs positive for FITC-conjugated ovalbumin was assessed on a BD FACSCalibur flow cytometer counting 2×10^4^ cells/sample [32].

### Killing Assay

IgG-opsonized *E. coli* were allowed to attach to PMN at 4°C for 30 min. Nonadherent bacteria were removed by washing with PBS. The samples were split into two aliquots and incubated either at 4 or 37°C on LB agar. To determine the number of bacteria initially attached to PMN (N4), serial samples kept at 4°C throughout the experiment were prepared and plated in triplicate on LB agar. Similarly, serial samples incubated for 30 min at 37°C were prepared and plated on LB agar to determine the number of surviving bacteria after incubation at 37°C (N37) for 24 h. The percentage of bacteria killed by PMN was calculated as follows: the percentage of killing = 100×(1−N37/N4) [33].

### Assay for Intracellular Respiratory Burst Response of PMNs

Both 2′, 7′-dichloroﬂuorescin diacetate (DCFH-DA Molecular Probe) was used to detect hydrogen peroxide as described previously [34]. PMNs were resuspended in 1 ml of PBS (concentration 1×10^6^ cells/ml) and prewarmed to 37°C for 5–10 min. Dye was loaded by adding 10 µM of DCFH-DA (Sigma), followed by a further incubation at 37°C for 45 min. The cells were washed three times with cold PBS before being measured on a BD FACSCalibur flow cytometer counting 2×10^4^ cells/sample.

### Real-time RT-PCR for the Analysis of FcγR Expression on Porcine PMNs

The total RNA of PMNs was extracted by using TRIZOL reagents (Invitrogen), and cDNA was subsequently synthesized using an Oligo-dT primer (TAKARA) and standardized to 1000 ng of the total RNA per sample. To measure the expression of porcine FcγRs relative to GAPDH in PMNs Real-time RT-PCR, gene-specific primers were designed. The oligonucleotide sequences of the upstream and downstream primers used for these mRNA analyses were:- 5′-TGTACCACCAACTGCTTG-3′ and 5′-ATCACGCCACAGTTTCC-3′ for GAPDH, 5′-GCACAGGATTTATCGGGAAGA-3′ and 5′-CAAGCAACCACAGCCACAA-3′ for FcγRII, and 5′-ACAAGGCCTCCAGCTACTCAA-3′ and 5′-AGCAACAGCCAGCCCTTATA-3′ for FcγRIIIA.

Each real-time PCR reaction (20 µl volume) contained 10 µl SYBR Green Real-time PCR Master Mix (TAKARA), 0.3 µM gene-specific primers and 1 µl standardized template cDNA. Amplification conditions were as follows: 95°C for 5 min, followed by 40 cycles of denaturation at 95°C for 30 seconds, annealing at 56°C for 30 seconds, and elongation at 72°C for 30 seconds. After the final extension at 72°C for 10 minutes, a melting-curve analysis was performed to ensure specificity of the products. FcγR gene expression was normalized to that of GAPDH, and expression relative to the sample with the lowest expression was calculated by using the Δ2^−ΔΔCT^ method [35].

### Cytokine ELISA

The amounts of the TNF-α and IL-1β in the serum samples were quantified following the standard protocols of the enzyme-linked immunosorbent assay kits purchased from Biosource. Briefly, Porcine TNF-α or IL-1β Standard (concentration 1250 pg/mL) was 2-fold diluted to produce a dilution series, respectively. Standards, Control, and serum samples were added into the wells of microplate which pre-coated with monoclonal antibody specific for porcine cytokines and any porcine cytokines present was bound by the immobilized antibody. After washing away any unbound substances, an enzyme-linked polyclonal antibody specific for porcine cytokines was added to the wells. Following a wash to remove any unbound antibody-enzyme reagent, a substrate solution is added to the wells. The enzyme reaction yields a blue product that turns yellow when the Stop Solution is added. The intensity of the color measured is in proportion to the amount of porcine cytokines bound in the initial step. The sample values are then read off the standard curve.

### PRRSV Quantification by Real-time RT-PCR

Total RNA was isolated from the serum samples. Subsequently, 1000 ng of the total RNA of every sample was reverse-transcribed using an Oligo-dT primer (TAKARA). The primers used in this study were designed from the open reading frame 7 (ORF7) from North American strain BJ-4 sequence as follows: 5′-CCTCTAGCGACTGAAGATGA-3′ and 5′-TTATCCTCCCTGAATCTGACA-3′. A single plasmid was constructed containing portion of the sequence of ORF7. Plasmid DNA concentrations were measured by optical density using a spectrophotometer (NanoDrop, Thermo) and copy numbers were calculated using the following equation: Copies/µL = [(6.02×10^23^ copies) × (plasmid concentration g/µL)]/[(number of bases)×(660 Daltons/base)] [36]. Tenfold serial dilutions of the plasmid (10^6^ copies to 1 copy) were prepared to create standard curve for ORF7. SYBR Green RT-PCR experiments were then carried out according to the manufacturer’s protocol. After each cycle, the fluorescence of SYBR green bound to double-stranded DNA was measured. The crossing point or the cycle number at which the fluorescence of the sample exceeded that of the background was determined using the second derivative method [37].

### Neutralizing Antibodies Test

Serial 2-fold dilutions of serum inactivated at 56°C for 30 min were made in DMEM and 0.05 mL of each dilution was mixed with an equal volume of the virus diluent containing 200TCID_50_/0.05 mL of virus. The serum-virus mixtures were incubated at 37°C for 60 min, as indicated in each experiment. At the end of incubation each mixture was inoculated in 0.05 mL volumes into 96 well microtiter plates; 2 wells were used for each serum dilution. After virus was adsorbed at 37°C for 1 h, the cultures were washed once with PBS, and fed with 0.05 mL of DMEM, incubated in a CO2 incubater at 37°C for 72 h, and examined for CPE. The antibody titer was expressed as the reciprocal of the highest serum dilution which showed complete inhibition of CPE in at least one of the two wells. The virus diluent used was DMEM containing 10% of the complement, unheated normal guinea pig serum, unless otherwise stated, and DMEM containing PBS or heat-inactivated (56°C, 30 min) guinea pig serum instead of unheated complement were served as control virus diluents. Back titration of the virus was carried out each time to confirm that 200 TCID50 of virus were used. Each sample was run in duplicate, and the mean titer was calculated [38].

### Statistical Analysis

Data were subjected to one-way analysis of variance (one-way ANOVA). If the P value from the ANOVA was less than or equal to 0.05, pair wise comparisons of the different infection groups were performed by least significant difference test at a rejection level of a P value < 0.05. The software GraphPad Prism v5.0 was used to make the graphs.

## Results

### Clinical Symptoms of Pigs Post PRRSV HN07-1 or BJ-4 Inoculation

On day two post-inoculation, all of the pigs inoculated with PRRSV HN07-1 showed an increased body temperature (>40°C), and had marked clinical symptoms including depression, anorexia, rubefaction of skin and ears, respiratory distress, shivering and diarrhea. One of the five animals in this group died at 35 DPI, and lung hemorrhagic spots were seen. Animals infected with PRRSV BJ-4 exhibited the same but relatively weak symptoms than PRRSV HN07-1 infected pigs, and no animals died in this group. Pigs in the control group did not show any clinical symptoms and survived to the experimental period. The results were consistent with previous research [28].

### Down-regulation of Antibody-dependent Phagocytosis by PMNs from Pigs Infected with PRRSV

**Figure 1:**
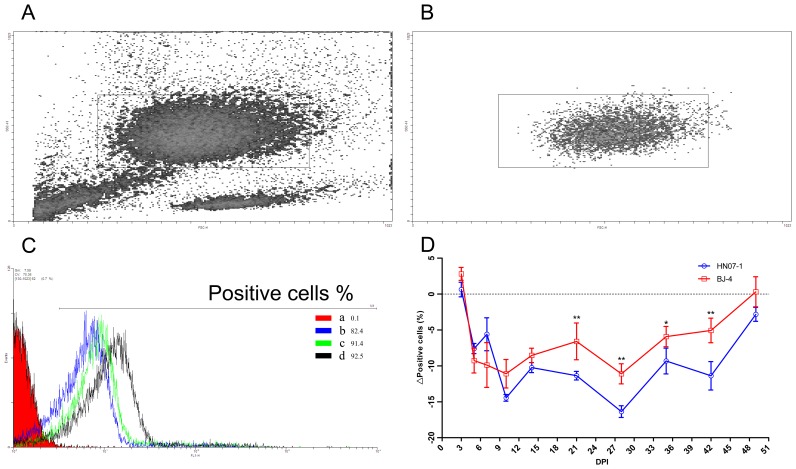
Immune complex adhesion assays. A. Scatter plot from a suspension containing erythrocytes and PMNs, the gate identifies PMNs. B. Only PMNs are indicated in the final preparation following lysis. C. Fluorescence intensity of gated neutrophils following incubation with FITC-labeled ovalbumin-Ig complex at 42 DPI. a, negative control, PMNs isolated from a control piglet, were incubated with medium alone. b, binding of IC by PMNs of a piglet pre-inoculated with HN07-1. c, binding of IC by PMNs of a piglet pre-inoculated with BJ-4. d, binding of IC by PMNs of a piglet of mock-infected control group. D. Time course of the down-regulation of IC surface binding by porcine PMNs following pre-inoculation with either PRRSV strain. The percentage of inhibition was calculated by the following formula: % Positive cells _inoculation_-%Positive cells _mock-infected control_. Results are shown as mean ± SE (n = 5). *, p<0.05. **, p<0.01.

**Figure 2:**
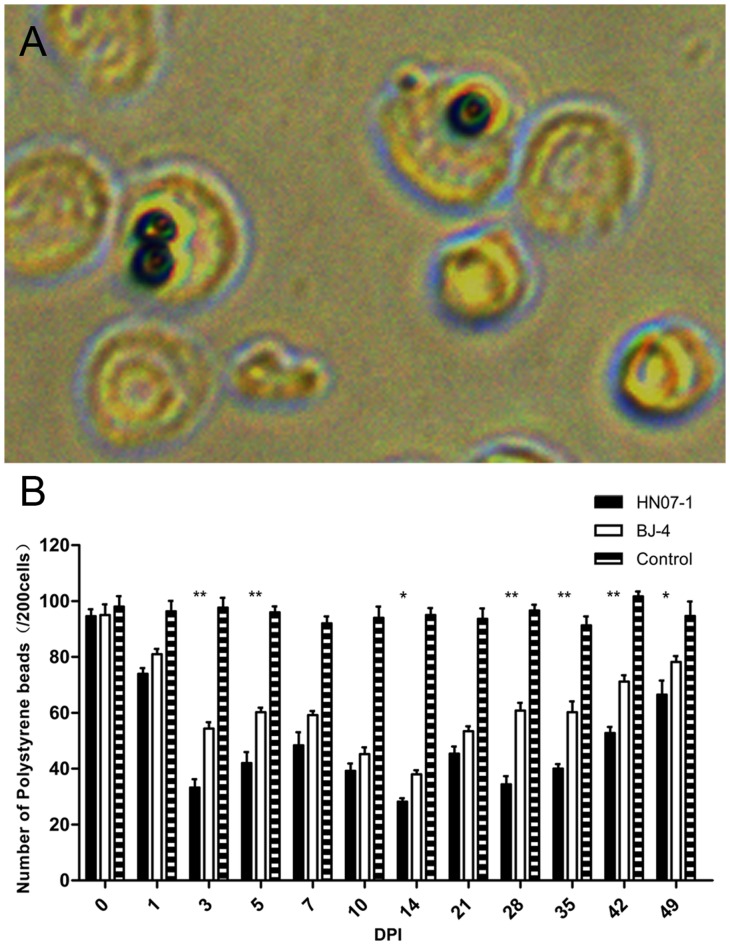
Phagocytosis (internalized particles per 200 cells) was quantified in serial samples after pre-inoculation with either PRRSV strains. Aliquots (1 ml) of a suspension of 1×10^6^ porcine PMNs were incubated with coated beads for 40 min at 37 °C. Phagocytosis of IgG-opsonized beads by PMNs is decreased after inoculation by either PRRSV strains. Error bars represent standard error of the mean from five animals. *, p<0.05. **, p<0.01.

Porcine IgG opsonized polystyrene beads, and FITC conjugated IC were prepared to analyze the effects of PRRSV infection on PMN phagocytosis. Flow cytometry indicated that the frequency of PMNs from PRRSV-infected pigs to phagocytose FITC conjugated IC was decreased by 5 DPI. PRRSV BJ-4 and HN07-1 both showed decreased phagocytosis, and this was greater with HN07-1 from 10 DPI to the end of the experimental period (Fig. 1). Microscopy indicated that the change in the phagocytic index of PMNs was in a similar tendency with the phagocytosis of IC, further confirming that the antibody-dependent phagocytosis of PMNs was down-regulated after PRRSV infection (Fig. 2).

### Ability of PMN to Kill*E.coli*

**Figure 3:**
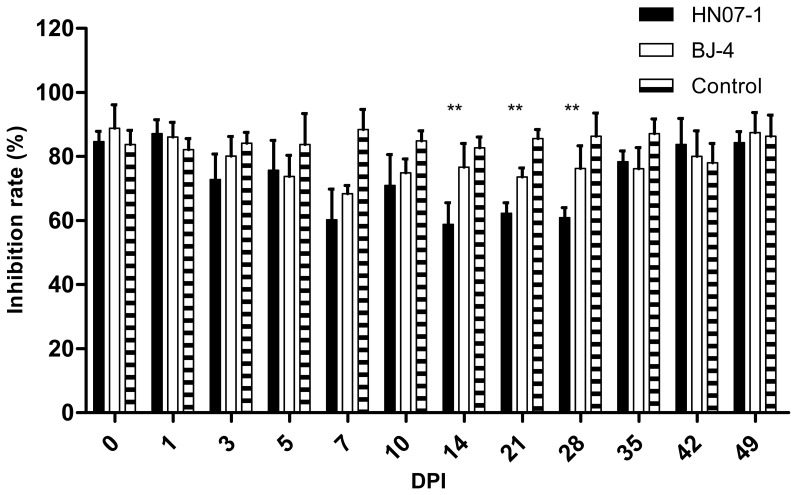
The viability of IgG-opsonized*E.coli* after incubation with PMN following PRRSV inoculation. IgG-opsonized *E.coli* were incubated with PMN at 4°C for 30 min. Cells were split over two aliquots and subsequently incubated for 30 min at either 4 or 37°C. Bacterial killing was determined from the difference between the numbers of viable bacteria initially attached to PMN (cells incubated at 4°C) and after bacterial phagocytosis (cells incubated at 37°C). The percentage of bacteria killed by PMN was calculated as follows: the percentage of killing = 100 × (1-N37/N4) Results are shown as mean ± SE (n = 5)*, p<0.05. **, p<0.01.

We evaluated the viability of IgG-opsonized *E.coli* after incubation with PMN. Bacterial killing was determined from the difference between the numbers of viable bacteria initially attached to PMN (cells incubated at 4°C) and after bacterial phagocytosis (cells incubated at 37°C). Using PMN of control group, we found IgG-mediated phagocytosis by PMN proved to induce efficient bacterial killing at an average of 83.7%. The ability of PMN to kill *E.coli* was decreased after inoculation with either PRRSV BJ-4 or HN07-1. HN07-1 shows a significantly lower killing ability level from 14 DPI to 28DPI than BJ-4 (Fig. 3). The result was similar with the test of PMN phagocytosis, confirming that the ability of PMN to clear invading pathogens was reduced by the inoculation with PRRSV.

### Effects of PRRSV on Respiratory Burst Response of PMNs

**Figure 4:**
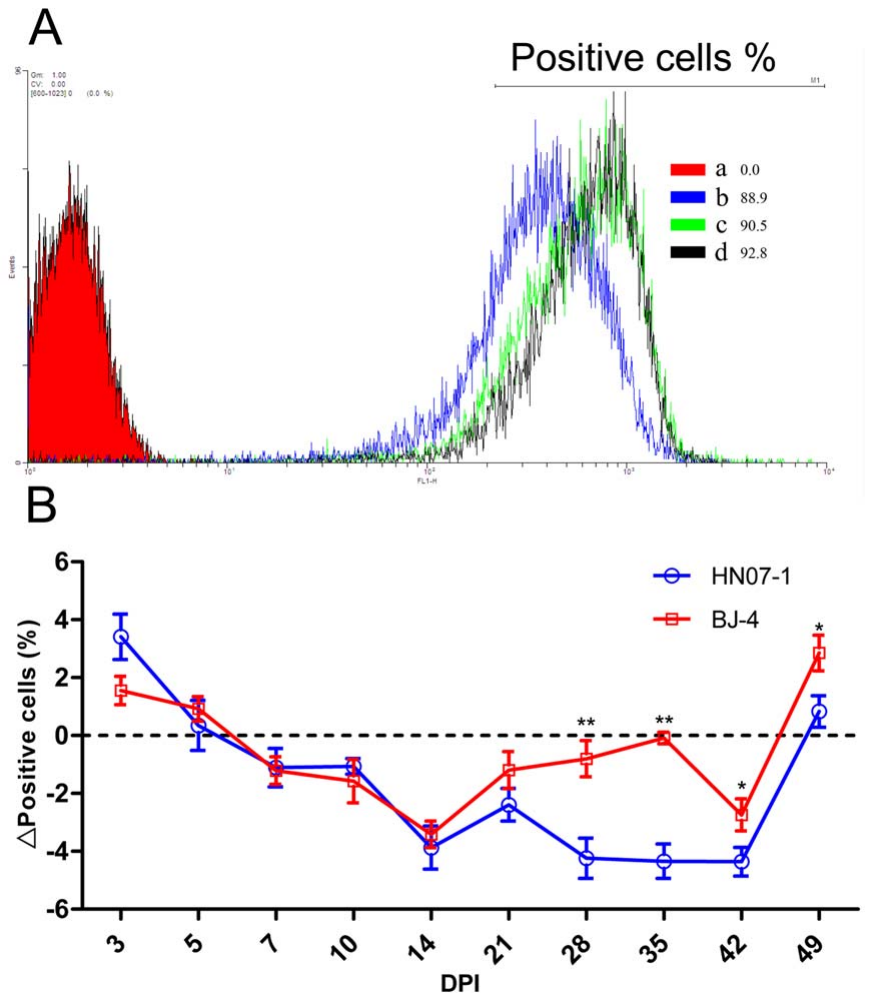
Effects of PRRSV on oxidative burst response in PMNs. Binding of DCFH-DA was measured by ﬂow cytometry at 14 DPI. a, negative control, PMNs isolated from a control uninoculated piglet, incubated with medium alone. b, binding of DCFH-DA by PMNs of a piglet inoculated with HN07-1. c, binding of DCFH-DA by PMNs of a piglet inoculated with BJ-4. d, binding of DCFH-DA by PMNs of a piglet from the mock-infected control group. B. Inhibition of PMN-induced oxidative burst following the pre-inoculation with either PRRSV strains. Error bars represent standard error of the mean from five animals. The percentage of inhibition was calculated by the following formula: %Positive cells _inoculation_- %Positive cells _mock-infected control_. Results are shown as mean ± SE (n = 5). *, p<0.05. **, p<0.01.

At first PMNs of piglets inoculated with either of the PRRSV isolates showed an increase in the respiratory burst response but after 3 DPI it fell and remained lower than the control (Fig. 4). DCFH-DA fluorescence also indicated a 3.87% (n = 5) reduction by 14 DPI. HN07-1 was a stronger inhibitor of the oxidative burst response of PMNs than BJ-4, although no significan difference was found until 28 DPI. Significant differences were observed between the two strains over 28 and 35 DPI with the inhibition by the PRRSV BJ-4 strain less than that of the HN07-1 strain (*p<0.01*). Statistical analysis using the least squares indicated that PRRSV inoculation may have inhibited the oxidative burst response in PMNs.

### Variation of Expression of FcγRs in PMNs of PRRSV-inoculated Piglets

**Figure 5:**
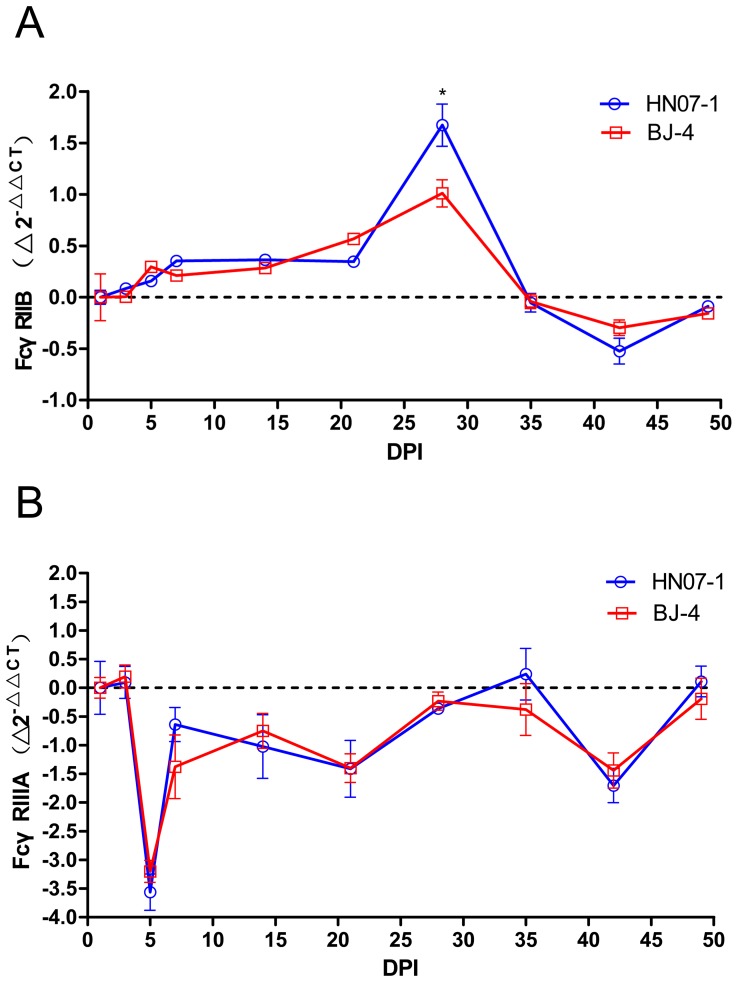
Time courses of the expression of FcγRs of PMNs after pre-inoculation with PRRSV strains. PMNs were isolated serially and FcγRs mRNA was measured by real-time PCR, and normalized relative to the expression of GAPDH. Results are shown as mean ± SE (n = 5). A. The expression of FcγRIIB. B. The expression of FcγRIIIA. The formula for calculating FcγRs modulation was Δ2^-ΔΔCT^: Δ2^-ΔΔCT^ = 2^-ΔΔCT^_ inoculation_-2^-ΔΔCT^_mock-infected control_.2^−ΔΔCT = ^2^-(CT, FcγR-CT, GAPDH) Time x-(CT, FcγR-CT, GAPDH) Time 0^. *, p<0.05. **, p<0.01.

To assess the effects of PRRSV inoculation on FcγRIIB and FcγRIIIA RNA expression of PMNs, we performed a quantitative analysis for the expression of mRNA of both FcγRIIB and FcγRIIIA by RT-PCR. As shown in Fig. 4A, in the piglets inoculated with HN07-1 the inhibitory receptor, FcγRIIB expression in PMNs increased significantly (*p = 0.029*), reaching a maximum at 28 DPI, compared with controls (Fig. 5A). It is noteworthy that BJ-4 inoculation also induced a mild upregulation of FcγRIIB expression with a similar time course, with no significant difference with that of HN07-1 pre-inoculation.

In contrast the RNA expression of FcγRIIIA after inoculation of either of the PRRSV strains was dramatically reduced and reached the lowest point at 5 DPI (Fig. 5B). Furthermore, there was no significant difference between the groups inoculated with the different PRRSV strains.

### IL-1β and TNF-a Responses after PRRSV Infection

**Figure 6:**
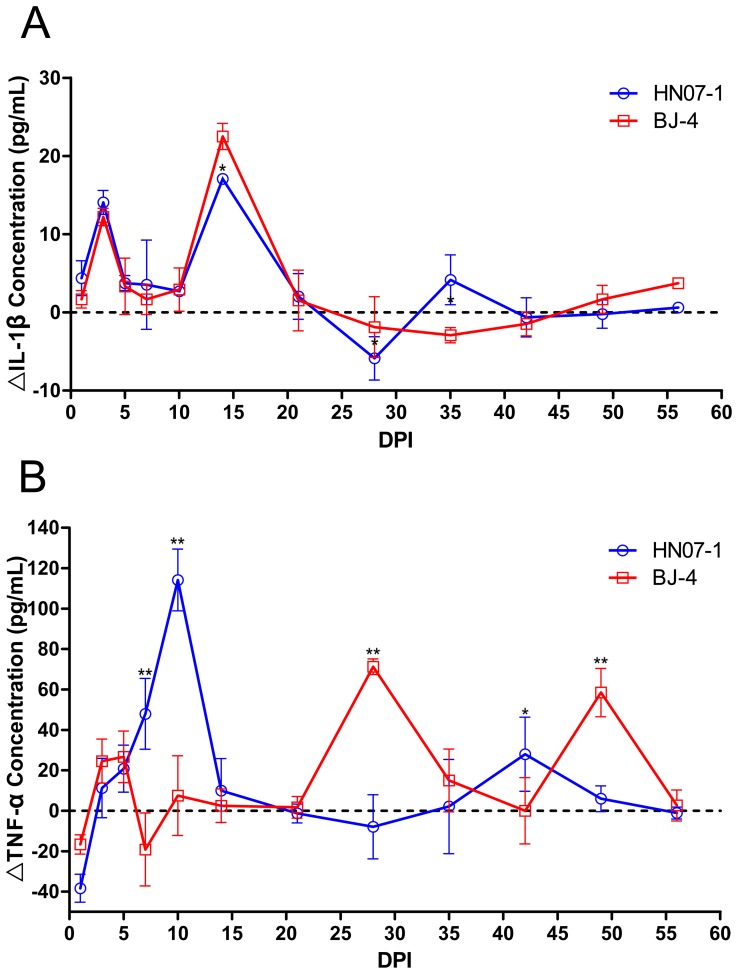
Serum levels of the pro-inflammatory cytokines IL-1β and TNF-α post PRRSV infection. Serum sample were obtained serially after inoculation by either PRRSV strains, and were assayed for A; IL-1β and B; TNF-α by ELISA. Means and SE (n = 5). *, p<0.05. **, p<0.01.

Serum levels of IL-1β show a rise at 3 and again at 15 days PI in both groups of infected animals (Fig. 6A). The response of the serum TNF-α level in the two infected groups (Fig. 6B) are noticeably different. After an initial fall in both groups at 3 DPI, there was a marked increase in the TNF-α level in the HN07-1 inoculated group over the controls, reaching a maximum at 10 DPI. However, BJ-4 was a weaker inducer of TNF-α than HN07-1, with a biphasic response showing maxima at 28 and 49 DPI (Fig. 6B).

### PRRSV Nucleic Acid in Serum

**Figure 7:**
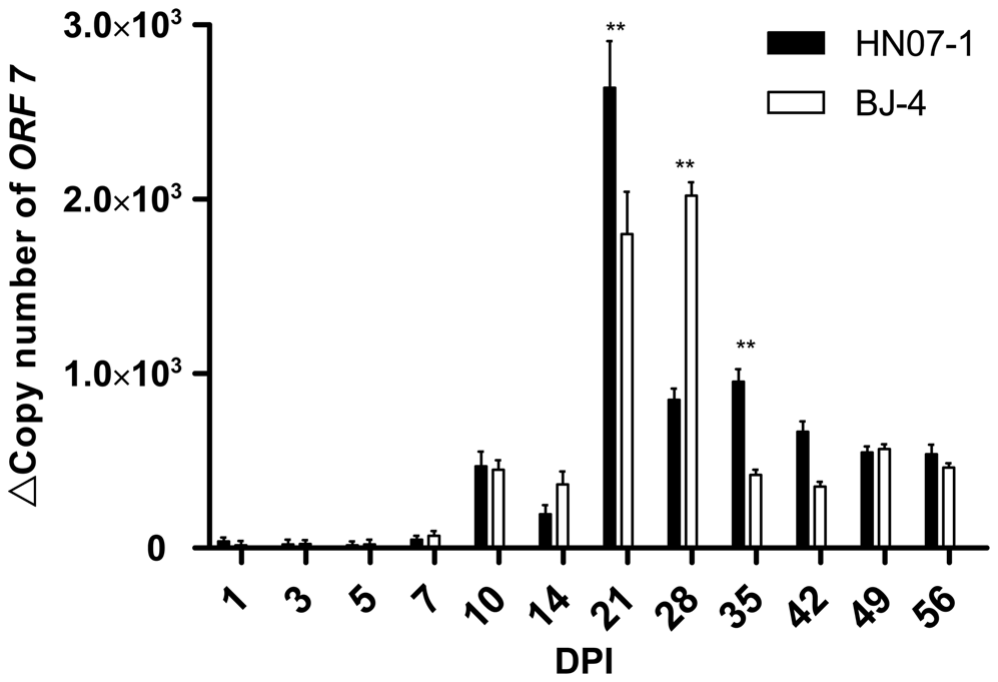
Results of the RT-PCR and SYBR Green ReTi RT-PCR for the detection of PRRSV in serum samples. Standard curves for ORF7 gene was constructed using 10-fold serial dilutions of the linearized plasmid ranging from 10^6^ copies to 1 copy per microliter. ORF7 mRNA levels in serum samples of different group were measured at each time point following PRRSV infection. Each cDNA sample was tested in the RT-PCR assay in triplicate. Error bars represent standard error of the mean from five animals. *, p<0.05. **, p<0.01.

All of the pigs in PRRSV-inoculated groups showed viraemia by the end of the study. The first positive PCR results were obtained in PRRSV-inoculated pigs (HN07-1 and BJ-4) at 3 DPI. Copy number of ORF7 increased, and peaked at 21 DPI (HN07-1 inoculation group) and 28 DPI (BJ-4 inoculation group) respectively. Significant differences were found from 14 DPI to 42 DPI between the two inoculated groups, suggesting that although HN07-1 inoculation has the capability to peak the virus load earlier (Fig. 7).

### NT Antibody Titers of Experimentally Infected Pigs

**Figure 8:**
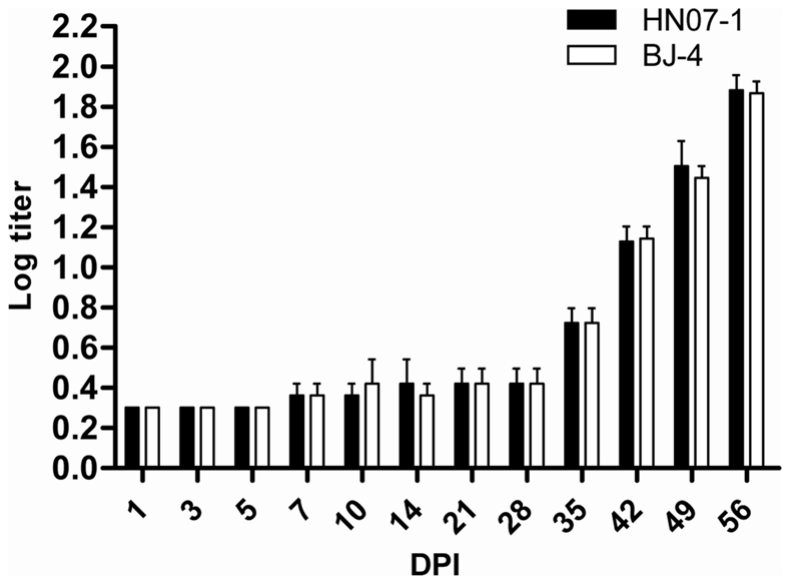
Neutralizing antibody titers of sera from the experimentally infected pigs. Serum sample were obtained serially after inoculation by either PRRSV strains, and were detected. Neutralizing antibodies were not detectable by seroneutralization tests in the first 4 weeks post-infection and were detectable to low titers by 35 DPI. Results are presented in average log titers (histogram bars) and shown as mean ± SE (n = 5) *, p<0.05. **, p<0.01.

Serum samples from experimentally infected pigs were tested for neutralizing antibody titer. Before experimental inoculation, all of the pigs were negative (neutralizing antibody titer:<4, log titer:<0.602 ) for antibody by NT test. Neutralizing antibodies in these animals were first detected at 35 DPI, then increased and peaked to a log titer of 1.881±0.075 (HN07-1 inoculation group) and 1.866±0.06 (BJ-4 inoculation group) at 49 DPI. No significant difference was observed between the NA tiers post BJ-4 or HN07-1 infection (Fig. 8).

## Discussion

Myeloid cells are key participants in host defense system and their FcγRs are highly significant mediators of their pinocytic and phagocytic activity in the clearance of foreign antigens [39]–[44]. FcγRIIB and FcγRIII are expressed on the surface of neutrophils, as well as monocytes, macrophages, eosinophils [12], [45]–[47]. Previous studies have indicated that FcγRIII is the main mediator of phagocytosis by human neutrophil [16], [32], [48]. However, conflicting reports about the role of FcγRIII role on neutrophils exist [49], [50]. Our finding supports that porcine FcγRIII plays an essential role in the phagoytosis by peripheral blood neutrophils [32], [48].

In our study the PMNs from piglets inoculated with either PRRSV strains showed a sharp down-regulation in IC-mediated phagocytosis. The expression of the inhibitory receptor FcγRIIB was increased and peaked at 28 DPI, whereas that of the activating receptor FcγRIIIA was decreased and reached the lowest point at 5 DPI in both PRRSV HN07-1 and BJ-4 infections, contributing to the decreased phagocytosis of PMNs and possibly to the suppression of immune responses. This result is consistent with previous investigations that suggest the decrease of FcγRIII on the surface of neutrophils is the major cause of the reduction in phagocytosis [15]–[17], [51]–[53]. Therefore, we concluded that the late upregulation of FcγRIIB and early depression of FcγRIIIA on PMNs following PRRSV inoculation suppressed the phagocytosis of PMNs.

Previous studies have shown that pro-inflammatory cytokines such as TNF could depress the expression of FcγR, particularly FcγRIII, in human PMNs, and FcγR expressions were not altered significantly by other cytokines, including IL-1α and β, IL-2, IL-3, IL-4, IL-6, G-CSF, GM-CSF and M-CSF [54]. Our study indicated an increase of TNF-α post PRRSV infection which may result in the depression of FcγRIII in porcine PMNs and the Ig-dependent phagocytosis of this cell was inhibited. Early studies have indicated that cytokine IL-1β mediates many protective and destructive effects of the immune response [55]. PMN are major producers of IL-1β and their contribution to IL-1β production could be important in early defense and healing [56]. In our study, the level of cytokine IL-1β upregulated during initial infection by PRRSV which may contribute to an enhanced virus level in the serum, because IL-1β has a minor effect on the length of virus replication, directly or indirectly, after PRRSV infection [57].

In addition we found that following infection the oxygen burst function of the PMNs was depressed. The production of oxygen and N free radicals is dependent on pinocytosis and phagocytosis but their production also further enhances phagocytic activity [58]. Such a reduction could therefore be an additional factor in the observed diminution of phagocytic activity. The interdependence of phagocytosis and free radical production is important in the killing of engulfed microorganisms. A reduction in the oxygen burst activity of the PMNs during PRRSV infection could partly explain the secondary infections in these animals [14].

PRRSV-infected pigs develop a strong and rapid humoral response but the initial antibodies do not confer protection [59]. Neutralizing antibodies (NAs) are consistently detected by day 28 PI or later for both European and American-type strains of PRRSV [60]–[62]. The early development of non-NAs may have a significant effect on the development of PRRS. It has been shown that non-NAs may enhance viral replication in alveolar macrophages, a phenomenon known as antibody dependent enhancement (ADE) [63]. In our study, neutralizing antibodies in the sera of infected pigs were not detectable by conventional neutralization tests (NTs) in the first 4 weeks post-infection. Even when NAs titers peaked at 56 DPI, it was still relatively low (log tier 1.866). No significant difference was found between the NAs titers post PRRSV HN07-1 or BJ-4 infection. The decrease of the copy number of PRRSV in serum after 28 DPI may attribute to the production of NAs post infection, and virus titers were suppressed with more production of NAs.

Finally, in all respects of cellar functions of PMN, the consequences of infection by the more highly pathogenic strain HN07-1 were greater on all of the parameters measured in this study. Whatever traits the PRRSV carries which make the host more susceptible to secondary infection [7]–[11], they appear likely to be more prominently expressed in PRRSV HN07-1. These observations may help to understand the mechanism of the increased susceptibility to secondary bacterial infection following PRRSV infection.
